# Adamantinoma of the ulna: A case report and literature review^☆^

**DOI:** 10.1016/j.ijscr.2024.109935

**Published:** 2024-06-22

**Authors:** Wazzan Aljuhani, Batool Alaskar, Mostafa Zolaly, Abdullah Alanazi, Abdulmalik Alanazi, Ali Assiri

**Affiliations:** a Department of Orthopedic Surgery, Ministry of the National Guard - Health Affairs, Riyadh, Saudi Arabia; b King Abdullah International Medical Research Center, Riyadh, Saudi Arabia; c King Saud Bin Abdulaziz University for Health Sciences, Riyadh, Saudi Arabia; d Department of Orthopedics, Prince Sultan Military Medical City, Riyadh, Saudi Arabia; e Department of Orthopedics, King Salman Medical City, Ministry of Health, Almadinah Almunawarah, Saudi Arabia; f Department of Pathology and Laboratory Medicine, Ministry of National Guard Health Affairs, Riyadh, Saudi Arabia; g College of Medicine, King Saud Bin Abdulaziz University for Health Sciences, Riyadh, Saudi Arabia

**Keywords:** Ulna, Adamantinoma, Tumor, En bloc resection, Kryptonite bone cement, Plate fixation

## Abstract

**Introduction and importance:**

Adamantinomas are rare, low-grade, malignant skeletal tumors accounting for only 0.33–0.48 % of primary malignant bone tumors.

**Case presentation:**

An 11-year-old boy with adamantinoma of the ulna was treated with en bloc resection, kryptonite bone cement, and plate fixation. The surgery resulted in marked pain relief and good functional recovery. No evidence of recurrence was observed for 5 years postoperatively, and the Musculoskeletal Tumor Society score was 93.

**Clinical discussion:**

This case of an 11-year-old previously treated for an aneurysmal bone cyst (ABC) illustrates the complexity of diagnosing adamantinoma, especially in atypical locations like the ulna. The successful use of en bloc resection and synthetic bone graft highlights the importance of accurate diagnosis and advanced surgical methods in achieving favorable outcomes in pediatric orthopedic oncology.

**Conclusion:**

Ulnar adamantinomas are rare and can be difficult to diagnose. The successful treatment of this tumor, as described in this case report, can help guide the management of similar cases in the future.

## Highlights

• The use of autogenous cancellous bone grafts is the current gold standard for the reconstruction of bony defects after resection.
• Synthetic grafts have the additional benefits of limitless availability and easy sterilization and storage In the present case, calcium triglyceride (kryptonite) was used as graft material.
• This patient had excellent outcomes 5 years after reconstruction with kryptonite, which adds further evidence to the durability of this material.

## 1 Introduction

Adamantinomas are rare, low-grade, malignant skeletal tumors accounting for only 0.33–0.48 % of primary malignant bone tumors [1,8]. They are considered osteolytic and locally aggressive, with 90 % of the cases occurring in the tibial diaphysis, followed by the forearms, hands, and feet [6,8]. Only a few cases of ulnar involvement have been reported in literature. These patients usually present with localized swelling lasting for weeks to months, with or without pain. On radiographs, adamantinomas appear as eccentric, well-circumscribed, lytic lesions with sclerotic borders, cortical thinning, and little periosteal reaction. They are usually treated with wide surgical excision [2]. Herein, we present the case of an adolescent boy with an adamantinoma of the ulna treated with en bloc resection, kryptonite bone cement, and plate fixation, with good outcomes 5 years postoperatively.

The work has been reported in line with the SCARE criteria [12].

## 2 Case Report

Consent was obtained from the patient for publication of this case report and the accompanying images. An 11-year-old boy was referred to our clinic because of a suspicious lesion in the left distal ulna, which was observed on radiographs. The patient complained of hyperesthesia, including increased sensitivity to pain in the same region. Five years previously, he had been diagnosed with an aneurysmal bone cyst (ABC) of the left distal ulna. He had undergone multiple surgeries, including incisional biopsy, curettage, bone grafting, and plate fixation. These procedures were performed at a different facility that did not have an orthopedic oncologist.

On physical examination at our clinic, the patient had hyperesthesia and tenderness at the left forearm along the ulnar nerve distribution. The ulna was deformed, resulting in limited pronation and supination. Radiography revealed a lytic lesion at the distal aspect of the ulna. Considering the patient's history and clinical examination, the radiographic findings could have been due to bone graft lucency, ABC recurrence, or delay in graft consolidation. Given the patient's previous surgical history, to avoid further insult to the ulnar nerve, the condition was thoroughly explained to the patient and his parents. Close monitoring was recommended until clear progression of the lesion was observed.

**Fig. 1:**
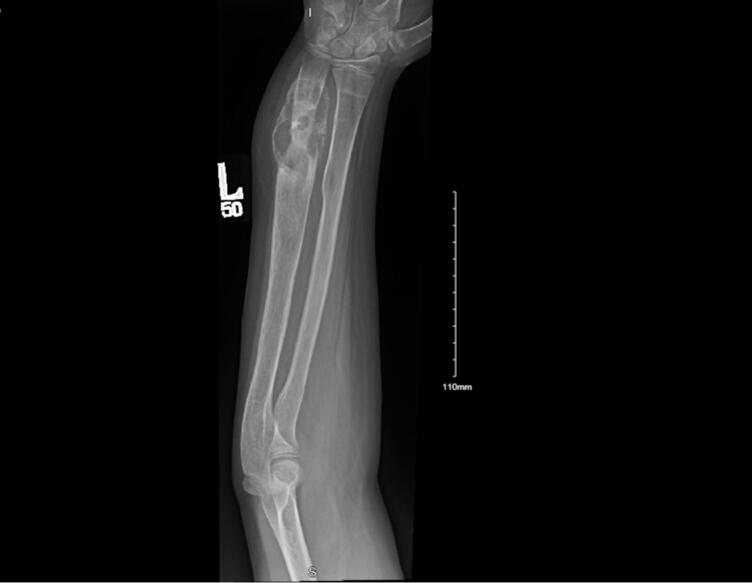
Radiograph obtained 1 year after the initial presentation; Anteroposterior view of the left forearm demonstrating a lytic lesion at the distal ulna.

**Fig. 2:**
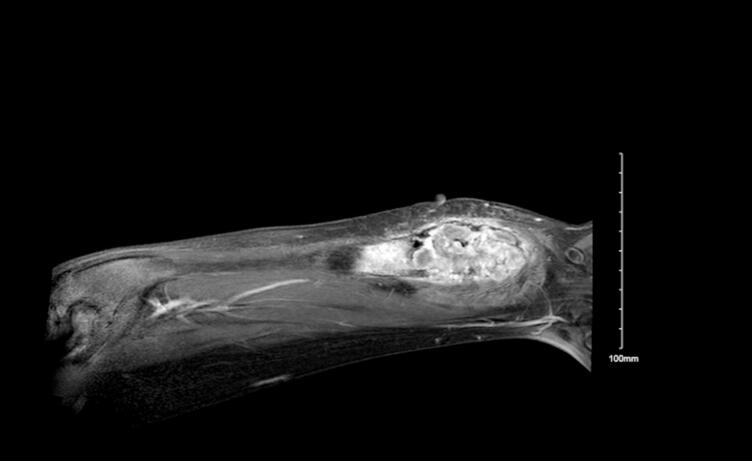
Magnetic resonance image obtained 1 year after the initial presentation. Coronal image demonstrating an expansive lesion involving the distal third of the left ulna with evidence of fluid-fluid level.

A year later, radiography demonstrated a clear progression of the lesion (Fig. 1). Magnetic resonance imaging (MRI) (Fig. 2) revealed an expansive lesion involving the distal third of the left ulna. The lesion had a ground-glass appearance with necrotic bone and fluid-fluid level. It appeared to be benign; however, malignancy could not be ruled out. Therefore, the patient was taken to the operating room to explore the lesion and obtain samples for histopathological analysis. Metastasis workup was also performed, including computed tomography of the chest, abdomen, and pelvis, all of which yielded negative results. After obtaining histological evidence, the patient and his parents were counseled on his new diagnosis and the need for more aggressive treatment. They were offered two options: resection and bone allograft with or without vascularized fibula autograft reconstruction as a definitive surgical option, or synthetic bone cement and plate fixation as a temporary solution until the final pathology results were obtained. They opted for the latter.

### 2.1 Histopathological findings

**Fig. 3:**
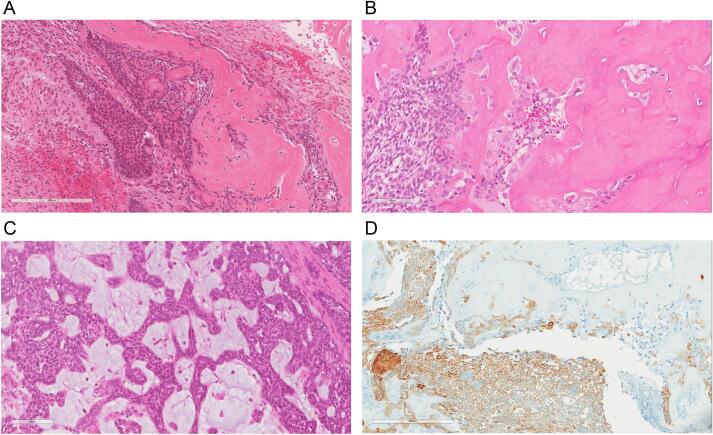
Histopathological findings; (A) Islands of classic adamantinoma with peripheral palisading and central areas of keratinization; (B) Adamantinoma cells with their stellate appearance and scalloping bone; (C) Trabecular growth pattern; (D) Positive immunostaining for keratin AE1/AE3.

Microscopic examination revealed a classic adamantinoma with a prominent epithelial component. The tumor exhibited nesting, trabecular, and sheeting patterns with peripheral palisading and central stellate cells (Fig. 3A–C). Immunohistochemically, co-expression of pan-cytokeratin (clone AE1/AE3), P63 (clone 4A4), and vimentin (clone V9) was observed (Fig. 3D).

### 2.2 Surgical procedure

**Fig. 4:**
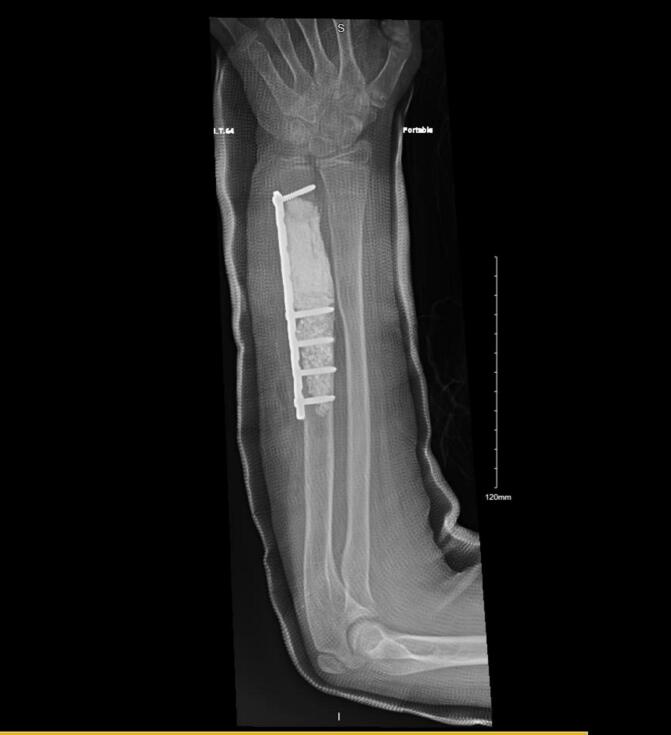
Radiograph obtained immediately after the surgery; Anteroposterior view of the left forearm demonstrating the cement, plate, and screw fixation after lesion resection.

The patient was placed in the supine position. General anesthesia was induced, and the patient was prepped and draped in a sterile manner. A sterile tourniquet was applied to the proximal region of the left arm. The operation was initiated by utilizing the previous incision on the distal ulna. The ulnar nerve was intact and protected in the index procedure and in the revision procedure, during the procedure the ulnar nerve was released from scar tissue and protected throughout the procedure and was not involved in the tumor. The entire lesion was observed after approaching the bone. En bloc resection was performed after proper MRI templating to achieve a negative margin. All margins were tumor-free after lesion resection, and the gap resulting from the resection was filled with kryptonite bone cement. The patient's ulna exhibited a positive variance, and osteotomy was performed to correct the deformity. Subsequently, plates and screws were used to fix the ulna (Fig. 4). Closure was performed in layers, and a sterile dressing was applied. The patient was discharged on postoperative day 5 with an elbow back-slab and a plan for thorough monitoring as an outpatient at our clinic.

### 2.3 Postoperative course

**Fig. 5:**
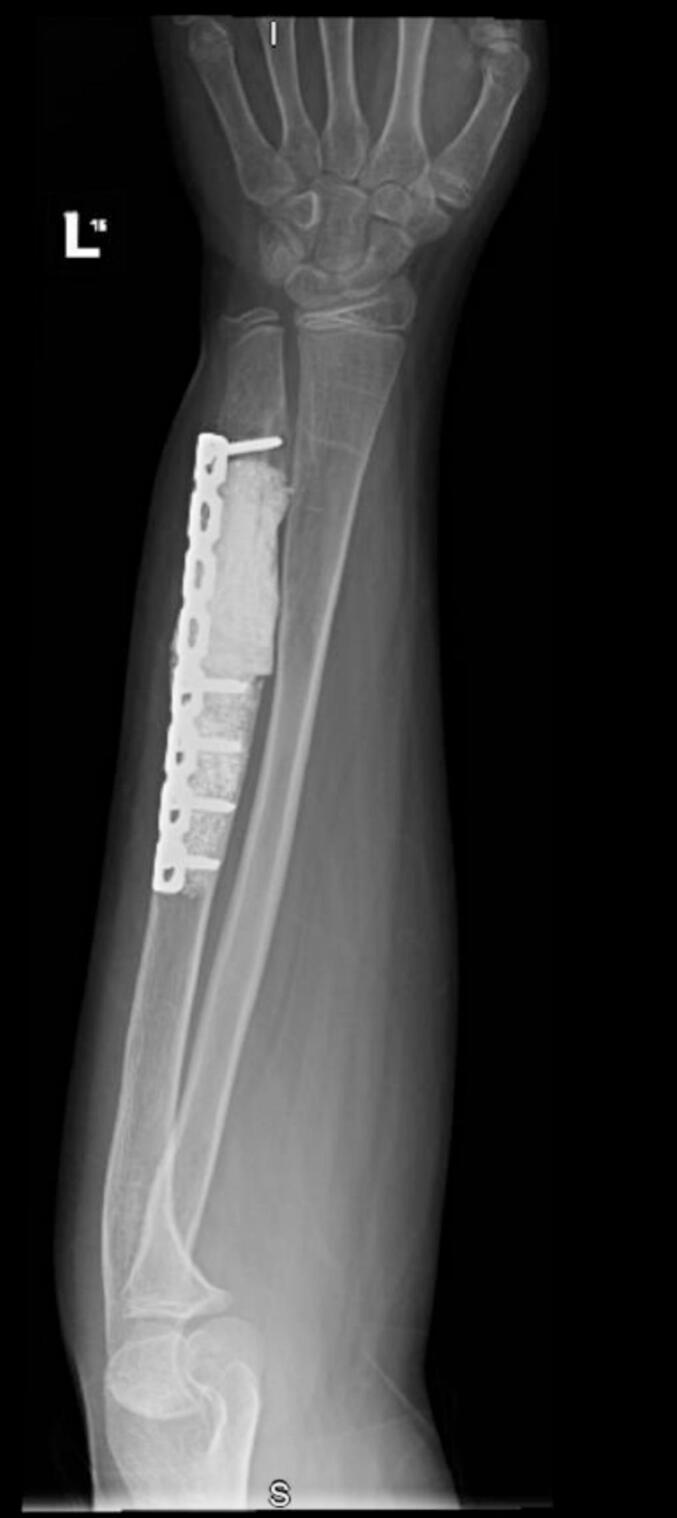
Radiograph obtained 5 years postoperatively; Anteroposterior view of the left forearm demonstrating stable cement, plate, and screw fixation at 5 years after lesion resection.

The patient was followed up at the outpatient clinic. He continually reported good functional outcomes at 2 weeks, 1 month, and 6 months postoperatively. During the 1-year follow-up, the pain and range of motion improved. The patient was satisfied with the outcomes and refused any further surgeries for reconstruction. No evidence of recurrence was observed 5 years postoperatively (Fig. 5), and the Musculoskeletal Tumor Society score was 93.

## 3 Discussion

We present the case of an adolescent boy with adamantinoma of the ulna, who was treated with en bloc resection, kryptonite bone cement, and plate fixation. The patient demonstrated good outcomes 5 years postoperatively. This case is unique because the patient had a lesion that presented in an unusual location following an initial diagnosis of ABC. Long-bone adamantinomas are rare musculoskeletal neoplasms. They are usually of unknown origin and typically contain epithelial and fibrous components [8]. The vast majority of these lesions occur in the tibia and anterior midshaft of the fibula (diaphysis), with 80 % occurring in the tibia alone [1,8]. They are slow growing tumors, and this accounts for their prolonged clinical course and delayed presentation. Symptoms can include pain, local swelling, or deformity [9]. Radiographically, they most frequently appear as well-circumscribed, locally expanded, intramedullary, osteolytic lesions with cortical thinning and minimal periosteal reaction [5,9]. On gross examination, adamantinomas often appear lobulated, and some contain cysts filled with blood or straw-colored fluid. Although findings on microscopic evaluations may vary widely, they typically comprise a fibrous stroma and exhibit epithelial palisading. Immunohistochemical analysis usually reveals epithelioid cells that are positive for antibodies against epithelial membrane antigens, cytokeratin, and vimentin. However, cytokeratin-negative adamantinomas have also been described [5,8]. Adamantinomas are typically treated surgically by wide en bloc resection and limb salvage surgeries where possible. The initial treatment is the most important factor for predicting outcomes and recurrence. Long-term disease-free survival can be expected in up to 100 % of patients when en bloc resection is performed. Marginal resection has led to recurrence in up to 31 % of reported cases [5]. Other factors associated with a more aggressive outcomes include male sex, short symptom duration, and presence of pain [5].

Adamantinomas have been hypothesized to originate from traumatically implanted epithelial elements, as almost all cases occur in the bones closest to the subcutaneous tissue. Furthermore, marked local trauma has been documented in 41 % of reported cases before diagnosis [3,4]. We believe that the trauma from the previous surgical treatment for the ABC might have contributed to the development of adamantinoma in our patient. Putnam et al. reported the first case of a classic adamantinoma with minor osteofibrous dysplasia-like foci and a secondary ABC [7]. This lends credence to the possibility of concurrent ABC and adamantinoma, initially considered by the authors. However, the difficulty in establishing the diagnosis of adamantinoma was possibly due to histological ambiguity or an inadequate specimen. In addition, the unusual location might have also contributed to the delayed diagnosis. The presentation observed in this case could also be attributed to inadequate initial treatment, which is considered an important factor for recurrence [5,8,9].

The use of autogenous cancellous bone grafts is the current gold standard for the reconstruction of bony defects after resection. However, autogenous cancellous bone grafts have several limitations. They are associated with the inability to obtain adequate substance, postoperative discomfort, and increased duration and cost of surgery [10]. This has led to innovations in research for reconstruction of bone defects using synthetic bone graft materials. Synthetic grafts have the additional benefits of limitless availability and easy sterilization and storage [10]. In the present case, calcium triglyceride (kryptonite) was used as graft material. Kryptonite is a porous, biocompatible polymer derived from castor oil. It hardens through a low-energy exothermic reaction, which causes less damage to the surrounding soft tissue [11]. It can also achieve rigid fixation and stability within 24 h, which allows early load transfer. This patient had excellent outcomes 5 years after reconstruction with kryptonite, which adds further evidence to the durability of this material [11].

In summary, we have reported a unique case of adamantinoma of the ulna that occurred following an ABC diagnosis. This report could provide more insight on the association between these two lesions. It may also add to the evidence regarding wide histological variations that might lead to delayed diagnosis. Furthermore, it emphasizes the importance of generating differential diagnoses as well as the need for extensive evaluation, even in lesions that occur in unusual locations. The successful treatment of this tumor, as described in this case report, can also help guide the management of similar cases in the future.

## Consent

Written informed consent was obtained from the patient parents/legal guardian for publication of this case report and accompanying images. A copy of the written consent is available for review by the Editor-in-Chief of this journal on request.

## Ethical approval

This study has been exempted from ethical approval by our institution.

## Funding

The authors received no funds for the research.

## Author contribution

Dr. Wazzan Aljuhani, Dr. Batool Alaskar, Dr. Mostafa Zolaly, Dr. Abdullah Alanazi, Dr. Abdulmalik Alanazi, Dr. Ali Assiri were involved in writing this case report

## Guarantor

Dr. Wazzan Aljuhani, Dr. Batool Alaskar, Dr. Mostafa Zolaly, Dr. Abdullah Alanazi, Dr. Abdulmalik Alanazi, Dr. Ali Assiri

## Research registration number

None.

## Conflict of interest statement

The authors declare no conflict of interest.
